# Supplementary material for: Modeling of DNA Damage Repair and Cell Response in Relation to p53 System Exposed to Ionizing Radiation
Source: Int J Mol Sci. 2022 Sep 26;23(19):11323. doi: 10.3390/ijms231911323 (PMC9569799; doi:10.3390/ijms231911323)
Supplement: Supplementary file 1 [file ijms-23-11323-s001.zip › ijms-1905291-supplementary.pdf]

Supplement materials

Table S1. Description of genes, mRNA and proteins in p53 system module

Symbol	Description
DSB	DNA double strand break
ATM	Kinase ATM (ataxia telangiectasia-mutated)
ATM_p	ATM phosphorylated at Ser1981 (ATM activated by DSB)
Wip1	Wild-type p53-induced phosphatase
$Wip1_{mRNA}$	Wip1 transcript
$Wip1_{gene}$	State of the Wip1 gene: active/inactive
SIAH1	Siah E3 Ubiquitin Protein Ligase 1
$SIAH1_p$	SIAH1 phosphorylated at Ser19
HIPK2	Homeodomain interacting protein kinase 2
p53	A tumor suppressor protein
$p53_{arrester}$	p53 phosphorylated at Ser15, Ser20, related to cell cycle arrest
$P53_{killer}$	p53 phosphorylated at Ser15, Ser20 and additionally at Ser46, related to apoptosis
$P53_{p46}$	p53 phosphorylated at Ser46 only
Mdm2	Proto-oncogene, main cellular antagonist of p53
$Mdm2_{gene}$	State of the Mdm2 gene: active/inactive
$Mdm2_{mRNA}$	Mdm2 transcript
$Mdm2_{cyt}$	Unphosphorylated Mdm2 in cytoplasmic
$Mdm2_{cyt\_2p}$	Mdm2 phosphorylated at Ser166 and Ser186 in cytoplasmic
$Mdm2_{nuc\_2p}$	Mdm2 phosphorylated at Ser166 and Ser186 in nucleus
$Mdm2_{nuc\_3p}$	Mdm2 phosphorylated at Ser166, 186 and additionally at Ser395 in nucleus
PI3K	Phosphoinositide 3-Kinase
PTEN	Phosphatase And Tensin Homolog, a multi-functional tumor suppressor
$PTEN_{gene}$	State of the PTEN gene: active/inactive
$PTEN^{mRNA}$	PTEN transcript
PIP2	Phosphatidylinositol 4,5-bisphosphate
PIP3	Phosphatidylinositol 3,4,5-triphosphate
Akt	AKT serine/threonine kinase
Akt_p	Akt phosphorylated at Thr308
Bax	BCL2 Associated X, Apoptosis Regulator
Bax_{gene}	State of the Bax gene: active/inactive
Bax_{mRNA}	Bax transcript
$Bclx_L$	B-cell lymphoma-extra large, an anti-apoptotic proteins of the Bcl-2 family
Bax : $Bclx_L$	complex of Bax and Bcl-x_L
Bad	Bcl2 associated agonist of cell death, a member of the Bcl-2 family
Bad_p	Bad phosphorylated at Ser75 and Ser99
BclxL : Bad	complex of Bcl-x_L and Bad
14-3-3	A family of small, acidic proteins that, through functioning adaptors,

	scaffolds, and chaperones, regulate a variety of intracellular signaling pathways.
Bad_p : 14-3-3	complex of Bad_p and 14-3-3
Caspase	Active caspase
ProCaspase	Inactive caspase
p21	A ell cycle inhibitor
$p21_{gene}$	State of the p21 gene: active/inactive
$p21_{mRNA}$	P21 transcript
CycE	E-type cyclins, limiting factor for G1 phase progression and S phase entry
p21 : CycE	Complex of p21 and Cyclin E
Rb1	Retinoblastoma (RB) Transcriptional Corepressor 1, negative regulator of the cell cycle and tumor suppressor
Rb1	Rb1 phosphorylated at Ser780
E2F1	E2F Transcription Factor 1,
Rb1 : E2F1	Complex of unphosphorylated Rb1 and E2F1
GADD45	Growth Arrest And DNA Damage-Inducible Protein
p38	Mitogen-Activated Protein Kinase P38
$p38_p$	p38 phosphorylated at Thr180/Tyr182
TGFβ	Transforming Growth Factor Beta

Table S2. Parameters of the p53 system module (If the reference is empty, the parameter is from Hat at al. [1])

Symbol	Value	Description	Reference
a1	3×10^{-10} s^{-1}	activation of proCaspases by Bax	
a2	1×10^{-12} s^{-1}	Caspases auto activation	
q0_pten	1×10^{-5} s^{-1}	PTEN gene spontaneous activation	
q0_wip1	1×10^{-5} s^{-1}	Wip1 gene spontaneous activation	
q0_mdm2	1×10^{-4} s^{-1}	Mdm1 gene spontaneous activation	
q0_bax	1×10^{-5} s^{-1}	Bax gene spontaneous activation	
q0_p21	1×10^{-5} s^{-1}	p21 gene spontaneous activation	
q1_pten	3×10^{-13} s^{-1}	PTEN gene activation induced by p53_killer	
q1_wip1	3×10^{-13} s^{-1}	Mdm2 gene act'n induced by p53_arrester	
q1_mdm2	3×10^{-13} s^{-1}	Wip1 gene act'n induced by p53_arrester	
q1_p21	3×10^{-13} s^{-1}	p21 gene act'n induced by p53_arrester	
q1_bax	3×10^{-13} s^{-1}	Bax gene act'n induced by p53_killer	
q2	3×10^{-3} s^{-1}	Inactivation of Mdm2, Wip1, p21 PTEN and Bax	
s1	0.1 s^{-1}	Wip1 mRNA synthesis	
s2	0.3 s^{-1}	PTEN mRNA synthesis	
s3	0.1 s^{-1}	Mdm2 mRNA synthesis	
s4	0.3 s^{-1}	Bax mRNA synthesis	
s5	0.1 s^{-1}	p21 mRNA synthesis	
t1	0.1 s^{-1}	Translation of Wip1	

t2	0.1 s^{-1}	Translation of PTEM	
t3	0.1 s^{-1}	Translation of Mdm2	
t4	0.1 s^{-1}	Translation of Bax	
t5	0.1 s^{-1}	Translation of p21	
s6	300 s^{-1}	p53 synthesis	
s7	30 s^{-1}	proCaspases synthesis	
s8	30 s^{-1}	HIPK2 synthesis	
s9	30 s^{-1}	Cyclin E synthesis induced by E2F1	
s10	3 s^{-1}	Spontaneous synthesis of Cyclin E	
p1	3×10^{-4} s^{-1}	ATM phosphorylation due to the presence of IR-induced DNA DSB	
p2	1×10^{-8} s^{-1}	SIAH1 phosphorylation by ATM_p	
p3	3×10^{-8} s^{-1}	p53 phosphorylation by ATM_p at Ser15 and Ser20	
p4	1×10^{-10} s^{-1}	p53 arrester phosphorylation by HIPK2 at Ser46	
p5	1×10^{-8} s^{-1}	Mdm2_cyt phosphorylation	
p6	1×10^{-8} s^{-1}	Mdm2_nuc_S116_S186 by ATM_p at Ser395	
p7	3×10^{-9} s^{-1}	Bad phosphorylation by Akt_p	
p8	3×10^{-9} s^{-1}	PIP2 phosphorylation by PI3K	
p9	3×10^{-6} s^{-1}	Rb1 phosphorylation by Cyclin E	
p10	3×10^{-6} s^{-1}	Rb1 phosphorylation by Cyclin E in Rb1-E2F1 complex	
p11	1×10^{-10} s^{-1}	P53 phosphorylation by HIPK2 at Ser46	
p12	1×10^{-9} s^{-1}	Akt phosphorylation by PIP3	
d1	1×10^{-8} s^{-1}	ATM_p dephosphorylation by Wip1	
d2	3×10^{-5} s^{-1}	$SIAH1_p$ dephosphorylation	
d3	1×10^{-4} s^{-1}	Spontaneous dephosphorylation of p53 arrester	
d4	1×10^{-10} s^{-1}	P53 killer dephosphorylation of Ser46 by Wip1	
d5	1×10^{-4} s^{-1}	Spontaneous dephosphorylation of Mdm2_cyt_S166p_S186p	
d6	3×10^{-5} s^{-1}	Mdm2_nuc_S166p_S186p_S395p dephosphorylation of S395p by Wip1	
d7	3×10^{-7} s^{-1}	PIP3 dephosphorylation to PIP2 by PTEN	
d8	1×10^{-4} s^{-1}	Spontaneous dephosphorylation of Akt_p	
d9	3×10^{-5} s^{-1}	Spontaneous dephosphorylation of Bad_p	
d10	1×10^{-4} s^{-1}	Spontaneous dephosphorylation of p53 killer at Ser15 and Ser20	
d11	1×10^{-10} s^{-1}	p53_s46 dephosphorylation by Wip1	
d12	1×10^{4} s^{-1}	Rb1 dephosphorylation	
b1	1×10^{-5} s^{-1}	Binding of Bax and $BclX_L$	
b2	3×10^{-5} s^{-1}	Binding of Bad and $BclX_L$	
b3	3×10^{-3} s^{-1}	Binding of Bad_p and 14-3-3	
b4	1×10^{-5} s^{-1}	Binding of Rb1 and E2F1	
b5	1×10^{-5} s^{-1}	Binding of p21 and Cyclin E	

u1	1×10^{-3} s^{-1}	Unbinding of Bax and $BclX_L$ complex	
u2	1×10^{-3} s^{-1}	Unbinding of Bad and $BclX_L$ complex	
u3	1×10^{-3} s^{-1}	Unbinding of Bad_p and 14-3-3 complex	
u5	1×10^{-4} s^{-1}	Unbinding of Rb1-E2F1 complex	
u6	1×10^{-4} s^{-1}	Unbinding of p21-Cyclin E complex	
i1	1×10^{-3} s^{-1}	Mdm2_cyt_S166p_S186p nuclear import	
g1	3×10^{-4} s^{-1}	mRNA_Wip1 degradation	
g2	3×10^{-4} s^{-1}	mRNA_PTEN degradation	
g3	3×10^{-4} s^{-1}	mRNA_Mdm2 degradation	
g4	3×10^{-4} s^{-1}	mRNA_Bax degradation	
g5	3×10^{-4} s^{-1}	mRNA_p21 degradation	
g6	3×10^{-5} s^{-1}	PTEN degradation	
g7	1×10^{-13} s^{-1}	HIPK2 degradation driven by Mdm2_nuc_2p and SIAH1	
g8	3×10^{-4} s^{-1}	Wip1 degradation	
g9	1×10^{-4} s^{-1}	Bax degradation	
g10	1×10^{-5} s^{-1}	Spontaneous degradation of p53	
g101	1×10^{-5} s^{-1}	Spontaneous degradation of p53 arrester	
g11	1×10^{-2} s^{-1}	p53 degradation induced by Mdm2_nuc_S166p_S186p	
g12	1×10^{-13} s^{-1}	p53_arrester degradation induced by Mdm2_nuc_S166p_S186p	
g13	1×10^{-13} s^{-1}	p53_killer degradation induced by Mdm2_nuc_S166p_S186p	
g14	1×10^{-4} s^{-1}	Mdm2_cyt degradation	
g15	3×10^{-5} s^{-1}	Spontaneous degradation of phosphorylated Mdm2	
g16	1×10^{-4} s^{-1}	Mdm2_nuc_S166p_S186p_ S395p degradation	
g17	3×10^{-4} s^{-1}	proCaspases degradation	
g18	3×10^{-4} s^{-1}	Caspase degradation	
g19	3×10^{-4} s^{-1}	p21 degradation	
g20	1×10^{-4} s^{-1}	Cyclin E degradation	
kGADD45act	4×10^{-9} s^{-1}	GADD45 Activation / Production	[2]
kGADD45deg	1×10^{-5} s^{-1}	GADD45 Degradation	[2]
kp38phos	8×10^{-3} s^{-1}	p38 Phosphorylation	[3]
kp38dphos	0.1 s^{-1}	p38 Phosphorylation	[3]
kTGFbeta	0.1 s^{-1}	generating TGFbeta by p38p	[3]
M1	7 or 25	Michaelis-Menten constant in ATM phosphorylation due to DSBs	[1, 4]
M2	1×10^{5}	Michaelis-Menten constant in Rb1 dephosphorylation at Ser567	
M3	2×10^{5}	Michaelis-Menten constant in E2F1-induced Cyclin E synthesis	

Table S3. Description of the gene, mRNA and protein interactions in p53 system

Reaction	Rate→	Rate←
$Wip1_{gene}$(inactive)←→$Wip1_{gene}$(active)	q0_wip1 + q1_wip1 * $p53arrester^2$	q2
$Mdm2_{gene}$(inactive)←→$Mdm2_{gene}$(active)	q0_mdm2 + q1_mdm2 * $p53arrester^2$	q2
$p21_{gene}$(inactive)←→$p21_{gene}$(active)	q0_p21 + q1_p21 * $p53arrester^2$	q2
$PTEN_{gene}$(inactive)←→$PTEN_{gene}$(active)	q0_pten + q1_pten * $p53killer^2$	q2
Bax_{gene}(inactive)←→Bax_{gene}(active)	q0_bax + q1_bax * $p53killer^2$	q2
ATM←→ATM_p	p1 * DSB^2 / ($M1^2$ + DSB^2)	d1*Wip1
SIAH1←→$SIAH1_p$	p2*ATM_p	d2
Φ←→HIPK2	S8	g7 * (SIAH1_0 + Mdm2_nuc_S166S186p) * (SIAH1_0 + Mdm2_nuc_S166S186p)
Φ←→$Wip1_{mRNA}$	s1 * $Wip1_{gene}$/2	g1
Φ←→Wip1	t1 * $Wip1_{mRNA}$	g8
Φ←→p53	s6	g101+ g11 * $Mdm2\_nuc\_2p^2$
p53 arrester→Φ	g12 * $Mdm2\_nuc\_S166S186p^2$	-
p53 S46p→Φ	g13 * $Mdm2\_nuc\_S166S186p^2$	-
p53 killer→Φ	g13 * $Mdm2\_nuc\_S166S186p^2$	-
p53←→p53 arrester	p3*ATM_p	d3
p53 S46p←→p53 killer	p3*ATM_p	d3
p53 arrester←→p53 killer	p3*HIPK2	d4*Wip1
p53←→p53 S46p	p3*HIPK2	d4*Wip1
Φ←→$Mdm2_{mRNA}$	s3* $Mdm2_{gene}$/2	g3
Φ←→Mdm2_cyt_0p	t3 * $Mdm2_{mRNA}$	g14
Mdm2_cyt_S166S186p→Φ	g15	-
Mdm2_nuc_S166S186p→Φ	g16	-
Mdm2_cyt_0p←→ Mdm2_cyt_S166S186p	p5*Akt_p	d5

Mdm2_cyt_S166S186p→Mdm2_nuc_S166S186p	i1	-
Mdm2_nuc_S166S186p←→Mdm2_nuc_S166S186p_S395p	p6*ATM_p	d6*Wip1
Φ←→$PTEN_{mRNA}$	s2*$PTEN_{gene}$/2	g2
Φ←→PTEN	t2*$PTEN_{mRNA}$	g6
PIP2←→PIP3	p8*PI3K	d7*PTEN
Akt←→Akt_p	p12*PIP3	d8
Φ←→$p21_{mRNA}$	g5*$p21_{gene}$	g5
Φ←→p21	t5*$p21_{mRNA}$	g19
Φ←→CycE	s10 + s9 * $E2F1^2$ / ($E2F1^2$ + $M3^2$)	g20
p21+CycE←→p21:CycE	b5	u6
p21:CycE→Φ	g20	-
Rb1←→$Rb1_p$	p9*CycE	d12 / (M2 + $Rb1_p$)
Rb1+E2F1←→Rb1:E2F1	b4	u5
Rb1:E2F1→$Rb1_p$+E2F1	p10 * CycE	-
Φ←→Bax_{mRNA}	s4*Bax_{gene}/2	g4
Φ←→Bax	t4*Bax_{mRNA}	g9
Bax+$Bclx_L$←→ Bax:$Bclx_L$	b1	u1
Bax:$Bclx_L$→ $Bclx_L$	g16	-
Bad+$Bclx_L$←→Bad:$Bclx_L$	b2	u2
Bad:$Bclx_L$→Bad_p+$Bclx_L$	p7*Akt_p	-
Bad←→Bad_p	p7*Akt_p	d9
Bad+14-3-3←→Bad:14-3-3	b3	u3
Bad:14-3-3→Bad_p+14-3-3	d9	-
Φ←→proCaspase	s7	g17
proCaspase→Caspase	a1*Bax + a2*$Caspase^2$	-
Caspase→Φ	g17	-
Φ←→GADD45	kGADD45act * p21	kGADD45deg
p38←→$p38_p$	kp38phos * GADD45	kp38dphos
Φ→TGFβ	kTGFbeta*$p38_p$	-

Φ means empty set, no protein or no mRNA.

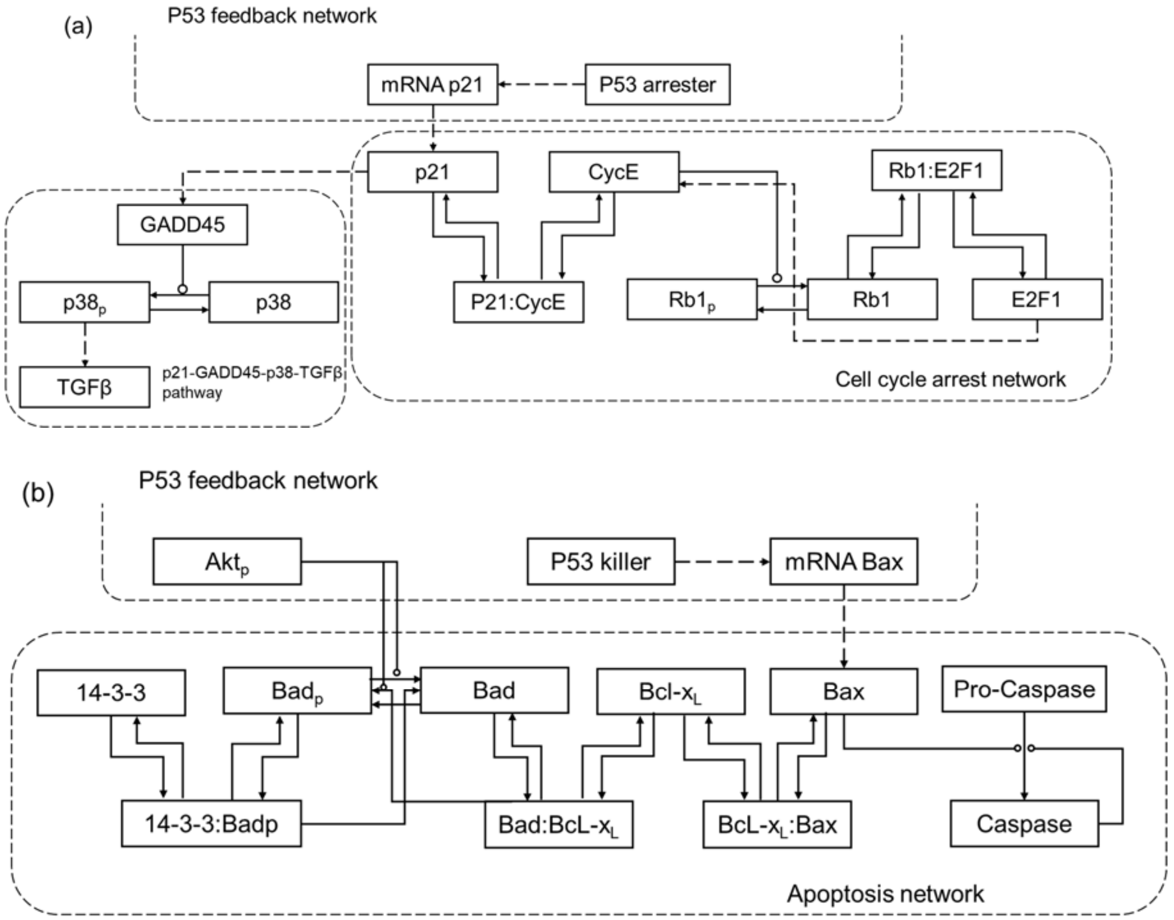

Figure S1. Detailed scheme of (a)the cell cycle arrest network and (b)apoptosis network in the p53 system. These two models are established based on the model of Hat et al. Arrow-headed dashed lines indicate positive transcriptional regulation, arrow-headed solid lines—protein transformation, circle-headed solid lines—positive influence or activation, hammer-headed solid lines—inhibitory regulation.

References

1. Hat, B.; Kochanczyk, M.; Bogdal, M. N.; Lipniacki, T., Feedbacks, Bifurcations, and Cell Fate Decision-Making in the p53 System. *PLoS Comput Biol* **2016,** 12, (2), e1004787.
2. Dolan, D. W.; Zupanic, A.; Nelson, G.; Hall, P.; Miwa, S.; Kirkwood, T. B.; Shanley, D. P., Integrated Stochastic Model of DNA Damage Repair by Non-homologous End Joining and p53/p21-Mediated Early Senescence Signalling. *PLoS Comput Biol* **2015,** 11, (5), e1004246.
3. Passos, J. F.; Nelson, G.; Wang, C.; Richter, T.; Simillion, C.; Proctor, C. J.; Miwa, S.; Olijslagers, S.; Hallinan, J.; Wipat, A.; Saretzki, G.; Rudolph, K. L.; Kirkwood, T. B.; von Zglinicki, T., Feedback between p21 and reactive oxygen production is necessary for cell senescence. *Mol Syst Biol* **2010,** 6, 347.
4. Ma, L.; Wagner, J.; Rice, J. J.; Hu, W.; Levine, A. J.; Stolovitzky, G. A., A plausible model for the digital response of p53 to DNA damage. *Proc Natl Acad Sci U S A* **2005,** 102, (40), 14266-71.
